# Anti-CGRP and Anti-CGRP Receptor Monoclonal Antibodies for Migraine Prophylaxis: Retrospective Observational Study on 209 Patients

**DOI:** 10.3390/jcm13041130

**Published:** 2024-02-17

**Authors:** Vittorio Schweiger, Paola Bellamoli, Francesco Taus, Leonardo Gottin, Alvise Martini, Marta Nizzero, Eleonora Bonora, Giovanna Del Balzo, Katia Donadello, Erica Secchettin, Gabriele Finco, Daniele De Santis, Enrico Polati

**Affiliations:** 1 Anesthesiology, Intensive Care and Pain Therapy Center, Department of Surgery, University of Verona, 37124 Verona, Italy; paola.bellamoli@aovr.veneto.it (P.B.); leonardo.gottin@univr.it (L.G.); alvise.martini@univr.it (A.M.); marta.nizzero@gmail.com (M.N.); eleonora.bonora@aove.veneto.it (E.B.); katia.donadello@univr.it (K.D.); erica.secchettin@univr.it (E.S.); enrico.polati@univr.it (E.P.); 2 Department of Diagnostics and Public Health, Section of Statistics, University of Verona, 37124 Verona, Italy; francesco.taus@univr.it; 3 Department of Diagnostics and Public Health, Section of Forensic Medicine, University of Verona, 37129 Verona, Italy; giovanna.delbalzo@univr.it; 4 Department of Medical Sciences and Public Health, University of Cagliari, 09124 Cagliari, Italy; gabriele.finco@unica.it; 5 Head and Neck Department, University of Verona, 37129 Verona, Italy; daniele.desantis@univr.it

**Keywords:** migraine, prophylaxis, monoclonal antibodies, calcitonin gene-related peptide (CGRP)

## Abstract

Background: Migraine is a neurological disorder characterized by attacks of head pain with prevalent unilateral localization, moderate to high intensity and specifically associated accompanying symptoms. Methods: In this retrospective observational study, we analyzed data regarding 209 patients who had previously been diagnosed with migraine and who were prescribed, between 2019 and 2022, subcutaneous injections of anti-CGRP monoclonal antibodies (mAbs) fremanezumab or galcanezumab or anti-CGRP receptors mAb erenumab regardless of the concomitant assumption of any other acute-phase or prophylactic migraine medication. Results: Regarding efficacy, in the 205 analyzed patients, the change from baseline in terms of MIDAS, HIT-6, MMDs and MAD scores was statistically significant for erenumab and galcanezumab, while for fremanezumab a statistical significance was not achieved likely due to the small sample size. In the treated population, 36 patients (17.5%) reported AEs (pain during injection, transient injection site erythema, nausea, constipation and fatigue). Only 5 patients (2.4%) discontinued the treatment for AEs while 15 patients (7.3%) left for lack of efficacy. Conclusions: this retrospective study comes out in favor of both significant efficacy and safety of anti-CGRP and anti-CGRP receptors mAbs in migraine patients. Further methodologically stronger studies are necessary to validate our observation.

## 1. Introduction

Migraine is a neurological disorder characterized by attacks of head pain with prevalent unilateral localization, moderate to high pain intensity and typical accompanying symptoms like nausea, vomiting, phonophobia and photophobia in different combinations and severity [1]. Beyond the typical pattern, there is a great heterogeneity of clinical presentations which often makes it difficult to perform a precise diagnosis and delays the start of correct treatment over time [2].

Based on the number of monthly attacks, migraine can be classified as episodic (EM, <14 migraine days per month) or chronic (CM, >15 days of headache per month for almost 3 months and at least 8 days of headache with migraine features) [1]. Among primary headaches, migraine is the second most frequent pattern after tension-type headache (TTH), with a prevalence of 14–15% among the general population [2]. According to some observations, migraine is the second major cause of disability worldwide after low back pain, and the first in the female population [3]. Moreover, migraine-related disability may lead to loss of productivity at work, while the cost effectiveness of investment in structured headache services to improve migraine treatment and work performance remains controversial [4]. To date, migraine pharmacological therapy includes attack medications often combined with prophylactic treatment and a range of non-pharmacological therapies [5]. Despite these options, optimal treatment and pain relief in migraine patients remain unsatisfactory [6]. New insights into migraine pathophysiology have been focusing on calcitonin gene-related peptide (CGRP), a neurotransmitter that emerged as one of the most relevant biological actors in the development of migraine [7]. The CGRP is released from activated trigeminal nerve terminals which binds to specific receptors on brain vessels and mast cells promoting vasodilation and mast cell degranulation, with subsequent neuroinflammation and nociceptor sensitization from several other neurotransmitters [7]. Over the past few years, new drugs acting on CGRP signaling pathways were developed and introduced on the market, in particular monoclonal antibodies (mAbs) targeted towards either CGRP or CGRP receptors [8]. The aim of this retrospective observational study was to evaluate the efficacy and safety of anti-CGRP (fremanezumab and galcanezumab) or anti-CGRP receptors (erenumab) mAbs in the prophylactic treatment of migraine patients referred to a tertiary headache center in Italy. 

## 2. Materials and Methods

### 2.1. Study Design

In this retrospective observational study, we analyzed the data routinely collected during the clinical practice in our headache center regarding patients aged ≥18 y previously diagnosed with migraine according to ICDH (International Classification of Headache Disorders) criteria 2018 of IHS (International Headache Society) [1] who, between 2019 and 2022, were prescribed subcutaneous injections of anti-CGRP mAbs fremanezumab (Ajovy^®^, TEVA Pharmaceuticals Europe B.V.,2031 GA Haarlem, The Nederland) or galcanezumab (Emgality^®^, Eli Lilly, Nederland B.V., 3528BJ Utrecht, The Nederland) or anti-CGRP receptors mAb erenumab (Aimovig^®^, Novartis Pharma GmbH, 90429 Nürnberg, Germany) regardless of the concomitant acute phase or prophylactic migraine medications. According to the latest AIFA (Agenzia Italiana del Farmaco) reimbursement prescription guidelines, in order to be enrolled, patients had to have a history of at least 8 days per month of disabling migraine, i.e., with a Migraine Disability Assessment Score (MIDAS) ≥ 11, and to have already been treated for at least 6 weeks with at least 3 different prophylactic medications (beta-blockers, anticonvulsants, tricyclic antidepressant, or in alternative botulinum toxin in case of CM) with lack of clinical response or the presence of known intolerance or contraindications to the proposed treatments [9]. The subcutaneous administration of mAbs was scheduled depending on the manufacturer’s instructions (erenumab 70 or 140 mg every 4 weeks, galcanezumab 240 mg loading dose followed by 120 mg once a month, fremanezumab 225 mg once a month (monthly dosing) or 675 mg every three months (quarterly dosing)). Treatments were prescribed based on each product’s availability, starting from erenumab (the first mAbs available on market in Italy since 2019), followed by galcanezumab and finally fremanezumab. 

### 2.2. Efficacy and Safety Assessment

The primary efficacy endpoint was the MIDAS score change from baseline (T0) to end of observation (T3, 13 months) [10]. The aim of the MIDAS questionnaire is to measure the impact of migraine on patients’ life, and it is based on five disability questions that focus on the time lost in terms of schoolwork or work for pay, household work or chores and family, social, and leisure activities. Scores from 5 to 10 indicate little to no disability, scores from 10 to 20 indicate moderate disability, and a score higher than 20 denotes severe disability. The MIDAS questionnaire has been validated in the Italian population [11]. Secondary efficacy endpoints were changes from baseline (T0) to end of observation (T3, 13 months) in HIT-6 (Headache Impact Test-6), MMD (Monthly Migraine Day) and MAD (Migraine Attack Duration in hours) scores. The HIT-6 score explores six items (pain, social functioning, role functioning, vitality, cognitive functioning, and psychological distress). The patient answers each of the questions using one of five possible choices (never, rarely, sometimes, very often, or always). The total HIT-6 score ranges from 36 to 78. A higher score indicates a greater impact of headache on daily life [12]. The HIT-6 score is widely used also in the Italian population for clinical and research purposes [13]. The MMDs (Monthly Migraine Days) and the MAD (Migraine Attack Duration in hours) scores are well known simple measures used to point out the efficacy of migraine treatment in clinical studies [14,15]. Data of MIDAS, HIT-6, MMDs and MAD were registered at baseline (T0) and after 4 months (T1), 7 months (T2) and 13 months (T3) from the start of treatment. Safety assessment included the frequency of treatment-related adverse events (AEs). The reasons that led to treatment discontinuation were also registered.

### 2.3. Data Collection and Statistical Analysis

We collected all data regarding mAbs erenumab, galcanezumab and fremanezumab treatments from the clinical documentation related to the institutional scheduled medical examination for headache patients. Demographic, medical, and clinical characteristics were summarized by descriptive statistics. We analyzed the data of the patients undergoing mAbs treatment of whom the MIDAS, HIT-6, MMDs and MAD scores at baseline visit and at least at one of the following medical assessments had previously been recorded. The MIDAS, HIT-6, MMDs and MAD questionnaires are administered to all headache patients referred to our center as part of routine clinical care. Categorical variables were expressed as numbers and percentages, while quantitative variables were expressed as medians and interquartile ranges (IQR). For paired data analysis, non-parametric Wilcoxon signed-rank test was used to compare differences in MIDAS, HIT-6, MMDs and MDA scores from baseline (T0) to end of observation (T3, 13 months). Statistical analysis was conducted using STATA statistical software, release 18 (StataCorp, College Station, TX, USA) setting the statistical significance at *p* < 0.05. All the graphs and related analyses were performed using GraphPad Prism software version 8.0 for Mac (GraphPad Software, San Diego, CA, USA).

## 3. Results

### 3.1. Study Population 

Between 2019 and 2022, mAbs erenumab, galcanezumab and fremanezumab were prescribed in our headache center to 209 patients suffering from migraine according to the ICHD criteria 2018 of IHS and to AIFA guidelines. Patient demographics and baseline characteristics are summarized in Table 1.

Among the three different mAbs treatments, patient demographics and baseline characteristics are summarized in Table 2.

**Figure 1:**
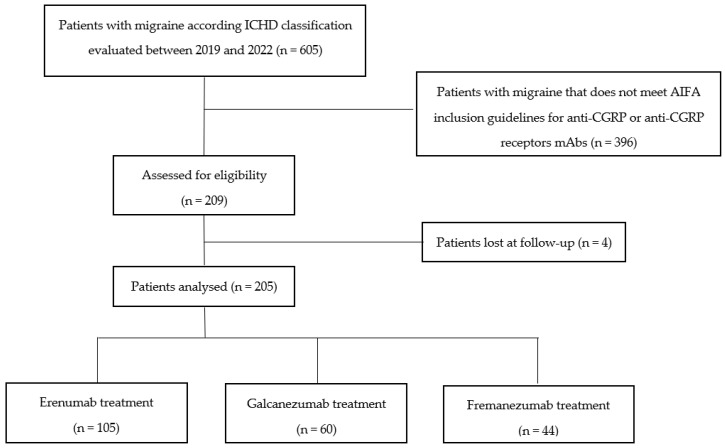
Flow diagram of the study population.

Among 209 included patients, 4 patients did not return to any medical evaluation after the treatment prescription and were therefore considered lost during follow-up, thus excluded from the final analysis. Flow diagram of the study population is represented in Figure 1. 

### 3.2. Efficacy

**Figure 2:**
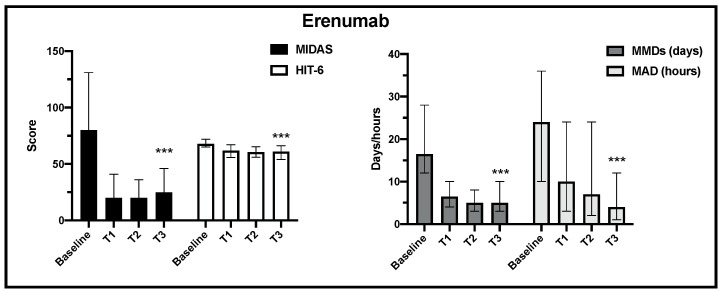
Change in MIDAS, HIT-6, MMD and MAD scores from baseline to the end of observation in erenumab treatment (T3) (*** *p* < 0.001).

**Figure 3:**
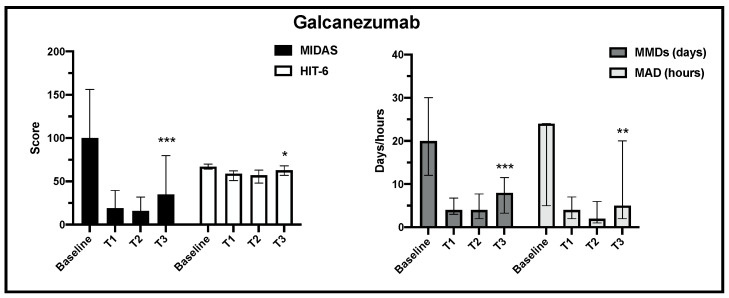
Change in MIDAS, HIT-6, MMD and MAD scores from baseline to the end of observation in galcanezumab treatment (T3) (*** *p* < 0.001, ** *p* < 0.01, * *p* < 0.05).

Regarding the primary and the secondary endpoints, in the 205 analyzed patients, the change over time in MIDAS, HIT-6, MMDs and MDA scores (median, IQR) was statistically significant for erenumab and galcanezumab treatment from baseline to the end of observation (Figure 2 and Figure 3).

**Figure 4:**
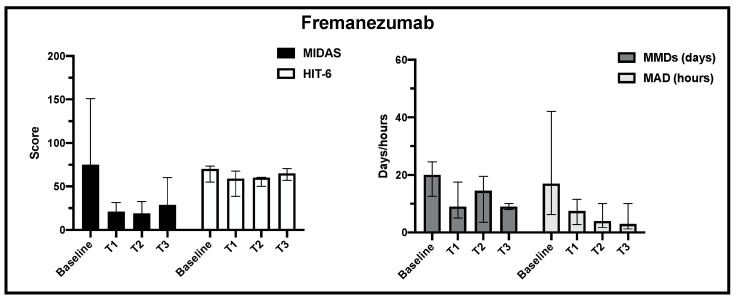
Change in MIDAS, HIT-6, MMD and MAD scores from baseline to the end of observation in fremanezumab treatment.

Regarding fremanezumab treatment, the MIDAS, HIT-6, MMD and MDA scores decreased significantly from baseline to T1 observation, but at the end of observation, due to the small sample size at this time point, statistical significance was not achieved (Figure 4).

All the data regarding time course of treatment in the three different mAbs are summarized in Table 3.

### 3.3. Treatment Discontinuation 

Overall, 24 patients (11.7%) discontinued mAbs treatment during the observation. Of these, 15 patients (7.3%) left for lack of efficacy, 2 patients (0.9%) for medical decision and 2 patients (0.9%) for personal choice. Only five patients discontinued the treatment for AEs (2.4%) (Table 4).

### 3.4. Safety

In the analyzed population, 36 patients (17.5%) reported AEs. Regarding the type of AEs, the most frequently reported were pain during injection (21 patients, 10.2%), and transient injection site erythema (18 patients, 8.7%). Other reported AEs were nausea (14 patients, 6.8%), fatigue (12 patients, 5.8%), constipation (12 patients, 5.8%), paresthesia in the extremities (2 patients, 0.9%) and transient hair loss (1 patient, 0.4%) (Table 5).

None of these patients discontinued the treatment, except for two patients who experienced constipation and one patient with paresthesia. In two patients (0.9%), cerebrovascular events during treatment were reported. In a 31-year-old male patient, a sudden worsening of headache 10 months after the start of treatment with erenumab led to an urgent brain MRI that showed a small left cerebellar ischemia then attributed to a focal ipsilateral vertebral artery dissection. The radiological finding disappeared within a few months with no negative outcome. In a 24-year-old female, the unusual appearance of persistent visual aura 6 months after the start of treatment with erenumab led to perform an urgent brain MRI that showed a small right frontal ischemia. Subsequent general examinations revealed a patent foramen ovale (PFO) with a right-to-left shunt. After PFO surgical closure, migraine improved significantly together with radiological findings. While the pathophysiology of these two events was not clearly attributable to mAbs, the treatments were discontinued in both patients as a precaution.

## 4. Discussion

Migraine pharmacological treatment is based on a different approaches depending on the burden of disease in each patient. To date, the use of pharmacological prophylaxis is recommended for patients with migraine attacks despite the use of symptomatic drugs while the number of headache days per month and headache severity to start a prophylactic treatment is still debated and may vary among different guidelines [5,16,17]. However, prophylactic treatment, based on different medications according to several approaches, showed consistent efficacy only for topiramate, while AEs related to this medication led to significant therapeutic non-adherence at 6 months after the start of treatment [18,19]. In this context, mAbs acting on CGRP or CGRP-receptors emerged as the most promising preventive treatment of migraine. The CGRP, a 37-amino-acid neuropeptide with strong properties of peripheral and central cerebral vasodilation, is released through the activation of cell bodies in the trigeminal ganglia. Substantial evidence indicates that the release of CGRP mediates the dilation of cerebral and dural blood vessels, the release of inflammatory mediators from mast cells, and the transmission of nociceptive information from intracranial blood vessels to the nervous system [7,20]. Its role in migraine development was confirmed by several findings like raised serum concentrations of CGRP during migraine attacks or relief of migraine by triptans which have been linked to the reduction or normalization in CGRP concentrations in blood samples [20]. The findings that selective CGRP receptor antagonists reduce vasodilation and neurogenic inflammation and confer clinical benefit in migraine further supported the crucial role of CGRP in migraine development [20]. To date, anti-CGRP or anti-CGRP receptors mAbs represent the most effective and safe treatment compared to all drugs prescribed for migraine prophylaxis [21]. This retrospective observational study confirmed this evidence. In our migraine population, the use of anti-CGRP or anti-CGRP receptors mAbs showed a relevant and long-lasting statistically significant improvement in all considered endpoints for erenumab and galcanezumab from baseline to the end of observation. All the endpoints’ scores decrease from baseline to the end of observation also for fremanezumab treatment, but due to the small sample size at this time point, statistical significance was not achieved. However, all the endpoints’ scores for fremanezumab treatment decreased statistically from baseline to the T1 observation point (3 months), where the number of patients was sufficient for statistical analysis. This reduction was similar to that highlighted for erenumab and galcazenumab at the same time point. Regarding different endpoints, the most consistent results for erenumab and galcanezumab were achieved for MIDAS, MMDs and MAD, while HIT-6 scores decreased less consistently, although still significantly. This was already noted in the literature, where HIT-6 and MIDAS appear to measure headache-related disability in a similar fashion but HIT-6 seems to be influenced by headache intensity more than the MIDAS, which is influenced more by headache frequency [22]. In fact, the “responder” definition for the HIT-6 total score was established in a ≥6-point decrease in a study on CM population [23]. Regarding treatment discontinuation, our results showed 11.7% of patients that left the treatment, most of all for lack of efficacy, while only five patients discontinued for AEs. Furthermore, the incidence of treatment-related AEs was low and limited to transient injection site pain and erythema, nausea, constipation, fatigue and paresthesia in the extremities. As previously stated, the majority of AEs did not lead to treatment discontinuation. All the available literature on mAbs erenumab, galcazenumab and fremanezumab reported a consistent reduction in MIDAS, HIT-6 and MMDs in the migraine population [24,25,26,27,28,29]. Moreover, the discontinuation from treatments was reported as limited, ranging from 4.5 to 22.5% according to different case series, indicating a good adherence to this prophylactic treatment (Table 6).

Indeed, even with the exclusion of studies with a short follow-up, anti-CGRP or anti-CGRP receptor mAbs seems to be better tolerated compared to topiramate, amitriptyline and propranolol, were discontinuation rate was estimated as 43.1%, 45.1% and 23%, respectively, between 16 and 26 weeks of treatment [30]. Our reported types of AEs were consistent with the other studies in the available literature, except for one patient with hair loss, while this AE was not certainly attributed to mAbs treatment [31]. Also, for the two patients that discontinued the treatment for cerebrovascular ischemic events, the correlation with concomitant mAbs treatment was not proved. Nevertheless, these events in our series deserve some in-depth analysis. To date, these AEs have rarely been reported in the literature, with a rate for erenumab of 0.02 per 1000 exposed patients, and have not been reported until now for the other mAbs available on the market [31]. There is no evidence from short- and long-term trials to indicate that mAbs targeting the CGRP pathway are associated with any increased risk of cerebrovascular ischemic events in migraine patients. Moreover, data from double-blind, placebo-controlled study on erenumab, with the aim of examining the rates of cerebrovascular AEs versus placebo, found no evidence of an association between treatment and this type of events [32]. Nevertheless, recent observations in a very large population of migraine patients confirmed that this headache was associated with an increased long-term risk of cerebrovascular events, both ischemic and hemorrhagic [33]. Although the underlying etiology for the association between migraine and cerebrovascular events remains unclear, several factors like hypercoagulable state, smoking, hyperlipidemia, hypertension and patent foramen ovale (PFO) with right-to-left shunt may explain this link [33]. To date, however, the patients with history or at risk of cerebrovascular events are excluded in Italy as a precaution from these treatments, as reported in the current AIFA Regulatory Statements [9].

## 5. Study Limitations

The study limitations are the retrospective nature of observation, reflecting the real life of a tertiary center for evaluation and treatment of migraine patients, and the relatively small number of patients still available for analysis at the end of the period of observation, particularly for the fremanezumab group. 

## 6. Conclusions

This retrospective observation confirmed the efficacy and safety of anti-CGRP and anti-CGRP receptors mAbs erenumab and galcazenumab in migraine patients, as stated in previous observations. Regarding fremanezumab, at the end of observation, the number of patients was insufficient to reach statistical significance, even though available results are encouraging. Further methodologically stronger studies will be necessary to validate our observation. 

## Figures and Tables

**Table 1:** Demographics and baseline characteristics of eligible population (209 patients).

Assessed for Eligibility	209
**Gender, n (%)**	
Female	179 (85.6%)
Male	30 (14.3%)
F:M ratio	6:1
Age, yr. *	51 (43–59)
**ICHD diagnosis n (%)**	
Chronic Migraine	148 (70.8%)
Episodic Migraine	61 (29.2%)
Migraine without aura	196 (93.8%)
Migraine with aura	13 (6.2%)
**Actual migraine attack drugs, n (%)**	
Triptans	75 (35.9%)
NSAIDs	44 (21.1%)

ICHD = International Classification of Headache Disorders, * = Median (IQR).

**Table 2:** Demographics and baseline characteristics of eligible population regarding the three different mAbs treatments (209 patients).

Assessed for Eligibility	209		
Treatment, n (%)	Erenumab, 105 (50.2%)	Galcanezumab, 60 (28.7%)	Fremanezumab, 44 (21.05%)
**Female, n (%)**	93 (88.6%)	52 (86.7%)	33 (76.7%)
**Male, n (%)**	12 (11.4%)	8 (13.3%)	10 (23.3%)
**F:M ratio**	8.7:1	6.5:1	3.3:1
Age, yr. *	50 (43–58)	52 (40–64)	53 (48–58)
**ICHD diagnosis n (%)**			
Chronic Migraine (CM)	78 (74.3%)	49 (81.7%)	21 (47.7%)
Episodic Migraine (EM)	27 (25.7%)	11 (18.3%)	23 (52.3%)
Migraine without aura	99 (94.3%)	58 (96.7%)	39 (88.6%)
Migraine with aura	6 (5.7%)	2 (3.3%)	5 (11.4%)
Headache scores *			
MIDAS (0–450)	80 (50–135)	107 (60–160)	65 (39–104)
HIT-6 (36–78)	68 (65–72)	58 (48–63)	66 (62–70)
MMDs (days)	17 (12–28)	16 (12–30)	15 (10–22)
MAD (hours)	24 (10–24)	24 (10–48)	10 (3–24)

MIDAS = Migraine Disability Assessment, HIT-6 = Headache Impact Test-6, MMDs = Monthly Migraine Days, MAD = Migraine Attack Duration, * = Median (IQR).

**Table 3:** Time course of endpoints regarding the three different mAbs treatments from baseline to end of observation. Endpoint reduction is expressed as percentage from baseline to T3.

TREATMENT		Time Course of Observation				Scores Reduction T3 vs. Baseline
**ERENUMAB**		**Baseline (n = 105)**	**T1 (n = 102)**	**T2 (n = 96)**	**T3 (n = 81)**	
**ENDPOINTS**	**MIDAS (0–450)**	80 (50–135)	25 (10–50)	20 (8–36)	25 (11–44)	68.7%
	**HIT-6 (36–78)**	68 (65–72)	62 (55–67)	60 (56–65)	61 (54–66)	10.3%
	**MMDs (days)**	17 (12–28)	5.5 (4–10)	5 (3–9)	7 (3–10)	58.8%
	**MAD (hours)**	24 (10–24)	10 (3–24)	6 (2–24)	4 (2–10)	83.3%
**GALCANEZUMAB**		**Baseline (n = 60)**	**T1 (n = 57)**	**T2 (n = 52)**	**T3 (n = 37)**	
**ENDPOINTS**	**MIDAS (0–450)**	107 (60–160)	18 (3–40)	18.5 (2.5–31.5)	35 (12–72)	67.2%
	**HIT-6 (36–78)**	66 (64–70)	58.5 (48–63)	60 (49.5–65)	63 (57–68)	4.54%
	**MMDs (days)**	16 (12–30)	4.5 (2–8)	4 (2–8)	8 (3.5–11)	50.0%
	**MAD (hours)**	24 (10–48)	5 (2–10)	2 (2–6)	5 (2–20)	79.2%
**FREMANEZUMAB**		**Baseline (n = 44)**	**T1 (n = 36)**	**T2 (n = 21)**	**T3 (n = 5)**	
**ENDPOINTS**	**MIDAS (0–450)**	65 (39–104)	10.5 (2.5–33.5)	9 (3–17)	29 (20–42)	55.4%
	**HIT-6 (36–78)**	66 (62–70)	55 (47–65)	60 (50–62)	65 (64–67)	1.5%
	**MMDs (days)**	15 (10–22)	4.5 (3–8)	4 (1–11)	9 (8–10)	40.0%
	**MAD (hours)**	10 (3–24)	3 (1–10)	4 (2–9)	3 (1.5–8)	70.0%

MIDAS = Migraine Disability Assessment, HIT-6 = Headache Impact Test-6, MMDs = Monthly Migraine Days, MAD = Migraine Attack Duration.

**Table 4:** Frequency of treatment discontinuation in the analyzed population (205 patients).

Treatment Discontinuation, n (%)	24	(11.7%)
Lack of efficacy	15	(7.3%)
Adverse Events (AEs)	5	(2.4%)
Personal choice	2	(0.9%)
Medical decision	2	(0.9%)

AEs = Adverse Events during treatment.

**Table 5:** Incidence and frequency of mAbs treatment-related AEs in the analyzed population. Some patients reported more than one AE. Percentages are related to whole analyzed population (205 patients).

Adverse Events during Treatment, n (%)	36 (7.5%)
Pain after injection	21 (10.2%)
Injection site erythema	18 (8.7%)
Nausea	14 (6.8%)
Fatigue	12 (5.8%)
Constipation	12 (5.8%)
Paresthesia	2 (0.9%)
Cerebrovascular events	2 (0.9%)
Hair loss	1 (0.4%)

**Table 6:** Study selection on anti-CGRP and anti-CGRP receptor mAbs treatment in patients with migraine.

Authors	Study Type	Patients (n)	mAbs Treatment	Follow-up (Months)	Dropout (n, %)	MIDAS Reduction (%)	HIT-6 Reduction (Points)	MMDs Reduction (%)
**Goadsby P.J. et al, 2020** [24]	Prospective	1890	Fremanezumab	12	396 (21%)	73.7%	8.4	62.5% (CM) 44.4% (EM)
**Lambru G. et al, 2020** [25]	Prospective	164	Erenumab	6	19 (12%)	n.a.	4	35%
**Torres-Ferrús M. et al. 2021** [26]	Prospective	155	Erenumab Galcanezumab	3	45 (22.5%)	65.2%	n.a.	47%
**Sette L. et al. 2022** [27]	Retrospective	90	Erenumab Fremanezumab	6	4 (4.5%)	n.a.	13	72.7%
**Iannone L.F. et al. 2022** [28]	Prospective	203	Erenumab Fremanezumab Galcanezumab	12	35 (19%)	74.2%	15	41.6%
**Schiano di Cola F. et al. 2023** [29]	Retrospective	152	Erenumab Galcanezumab Fremanezumab	6	n.a.	63.2%	n.a.	45.5%

MIDAS = Migraine Disability Assessment Score, HIT-6 = Headache Impact Test-6, MMDs = Monthly Migraine Days, n.a. = not available.

## Data Availability

Database is not available for data sharing. The institutional consent form administered to patients did not provide the silent agreement for data sharing.
